# 773 Inhalation Injury Severity Score on Admission Predicts Overall Survival in Burn ICU Patients

**DOI:** 10.1093/jbcr/irad045.248

**Published:** 2023-05-15

**Authors:** Ashley Flinn, Phillip Kemp Bohan, Catherine Rauschendorfer, Tuan Le, Julie Rizzo

**Affiliations:** Brooke Army Medical Center, San Antonio, Texas; Brooke Army Medical Center, San Antonio, Texas; United States Army Institute of Surgical Research, San Antonio, Texas; United States Army Institute of Surgical Research, San Antonio, Texas; Brooke Army Medical Center, La Vernia, Texas; Brooke Army Medical Center, San Antonio, Texas; Brooke Army Medical Center, San Antonio, Texas; United States Army Institute of Surgical Research, San Antonio, Texas; United States Army Institute of Surgical Research, San Antonio, Texas; Brooke Army Medical Center, La Vernia, Texas; Brooke Army Medical Center, San Antonio, Texas; Brooke Army Medical Center, San Antonio, Texas; United States Army Institute of Surgical Research, San Antonio, Texas; United States Army Institute of Surgical Research, San Antonio, Texas; Brooke Army Medical Center, La Vernia, Texas; Brooke Army Medical Center, San Antonio, Texas; Brooke Army Medical Center, San Antonio, Texas; United States Army Institute of Surgical Research, San Antonio, Texas; United States Army Institute of Surgical Research, San Antonio, Texas; Brooke Army Medical Center, La Vernia, Texas

## Abstract

**Introduction:**

Inhalation injury (II) is identified in about one-third of burn patients and is associated with increased morbidity and mortality. There are multiple scoring systems to grade II; however, no study has compared ability of these scoring systems to predict outcomes of interest such as overall survival. We hypothesized that II grades from each scoring system assessed would correlate strongly and that higher scores (representing higher grade II) would be associated with worse overall survival (OS).

**Methods:**

This was a prospective, observational study that included intubated burn patients who underwent fiberoptic bronchoscopy within 24 hours of admission. II was graded using three scoring systems: Abbreviated Injury Score (AIS), Inhalation Injury Severity Score (I-ISS), and Mucosal Score (MS). All scoring systems use a Likert-type scale (AIS: 0-4; I-ISS and MS: 0-3) with more severe IIs given higher scores. Agreement between scoring systems was assessed with Krippendorff’s Alpha (KA) >0.80 indicating high reliability. Multivariable analyses were used to determine variables independently associated with OS. Significance was set at p< 0.05.

**Results:**

99 patients were included in the analysis. Median percent total body surface area burned and Injury Severity Score (ISS) were 19.5% (interquartile range [IQR] 9-39%) and 25 (13-34), respectively. At admission, median AIS, I-ISS, and MS scores were 2 (1-3) for all scoring systems. Patients who died had higher overall injury burden than those who survived (Table 1). Additionally, patients who died had similar median admission AIS and MS scores, but higher I-ISS scores than those who survived (Table 1). There was strong correlation between the grade of II at admission using the three scoring systems (KA=0.85). On regression analysis, the only II scoring system independently associated with overall survival was I-ISS (score 3 compared to scores 1-2: OR 13.16, 95% CI 1.65-105.07; p=0.02).

**Conclusions:**

Grade of II on admission correlated well between scoring systems, but only I-ISS on admission was independently associated with OS. Progression of injury after initial assessment may contribute to the poor correlation between admission score and overall survival for IIs graded with AIS and MS. Repeated assessment and re-grading II after initial resuscitation may more accurately identify patients at increased risk for mortality.

**Applicability of Research to Practice:**

Grading II is common practice in Burn ICUs. This study demonstrates good reliability between the three most common II scoring systems utilized and that I-ISS on admission can be used to predict risk of mortality, which is useful prognostic information for both burn care providers as well as patients and their families.

